# Strategies to Overcome Intermediate Accumulation During *in situ* Nitrate Remediation in Groundwater by Hydrogenotrophic Denitrification

**DOI:** 10.3389/fmicb.2021.610437

**Published:** 2021-03-04

**Authors:** Clara Duffner, Anja Wunderlich, Michael Schloter, Stefanie Schulz, Florian Einsiedl

**Affiliations:** ^1^Chair of Soil Science, TUM School of Life Sciences Weihenstephan, Technical University of Munich, Freising, Germany; ^2^Research Unit Comparative Microbiome Analysis, Helmholtz Center Munich, Neuherberg, Germany; ^3^Chair of Hydrogeology, TUM Department of Civil, Geo and Environmental Engineering, Technical University of Munich, Munich, Germany

**Keywords:** nitrate pollution, hydrogen-oxidizing denitrification, nitrite accumulation, bioremediation, abiotic nitrite reduction

## Abstract

Bioremediation of polluted groundwater is one of the most difficult actions in environmental science. Nonetheless, the clean-up of nitrate polluted groundwater may become increasingly important as nitrate concentrations frequently exceed the EU drinking water limit of 50 mg L^–1^, largely due to intensification of agriculture and food production. Denitrifiers are natural catalysts that can reduce increasing nitrogen loading of aquatic ecosystems. Porous aquifers with high nitrate loading are largely electron donor limited and additionally, high dissolved oxygen concentrations are known to reduce the efficiency of denitrification. Therefore, denitrification lag times (time prior to commencement of microbial nitrate reduction) up to decades were determined for such groundwater systems. The stimulation of autotrophic denitrifiers by the injection of hydrogen into nitrate polluted regional groundwater systems may represent a promising remediation strategy for such environments. However, besides high costs other drawbacks, such as the transient or lasting accumulation of the cytotoxic intermediate nitrite or the formation of the potent greenhouse gas nitrous oxide, have been described. In this article, we detect causes of incomplete denitrification, which include environmental factors and physiological characteristics of the underlying bacteria and provide possible mitigation approaches.

## Introduction

Increased amounts of reactive nitrogen (Nr) and severe anthropogenic intervention in the global nitrogen cycle induce climatic change, cause biodiversity losses, and pose direct and indirect risks to human health (Fields, 2004; Galloway et al., 2008). In groundwater, the main Nr species is dissolved nitrate (NO_3_^–^) which leaches into the groundwater due to excessive use of chemical and organic fertilizers as well as leaking sewage (Zhu et al., 2019; Wild et al., 2020). The resulting NO_3_^–^ pollution of groundwater has been a severe global environmental problem since the 1970s (Rivett et al., 2008). Because groundwater infiltrates into rivers, lakes, and subsequently into coastal areas these ecosystems suffer from Nr-based eutrophication leading to toxic algal blooms and consequently anoxic “dead-zones” (Fields, 2004; Galloway et al., 2008) when natural attenuation processes fall short. A prominent example for coastal eutrophication is the Baltic Sea (Murray et al., 2019). The effect of eutrophication on urban lakes is also severe, as total-N and NO_3_^–^-N are one of the primary factors determining the algal community composition (Zhang et al., 2021). Additionally, in many regions where groundwater is used as a drinking water resource, NO_3_^–^ concentrations above the WHO recommended maximum of 50 mg L^–1^ require costly *ex situ* methods of NO_3_^–^ removal (Karanasios et al., 2010) or blending with less polluted water to ensure drinking water quality.

Denitrification, includes four main redox reactions from NO_3_^–^ (redox state + V), via nitrite (NO_2_^–^), nitric oxide (NO), and nitrous oxide (N_2_O) to atmospheric nitrogen (N_2_, redox state 0), each catalyzed by a different metalloenzyme (Bothe et al., 2007). Since the first reduction step from NO_3_^–^ to NO_2_^–^ is also performed in other metabolic pathways and gaseous N_2_O gas can already leave the ecosystem, in a strict sense denitrification includes only NO_2_^–^ and NO respiration (Zumft, 1997). Nonetheless, because NO_3_^–^ remediation aims at safely removing nitrogen from highly polluted aquatic systems, without releasing the greenhouse gas N_2_O, in this work complete denitrification signifies the reduction of NO_3_^–^ to N_2_. Only when the oxygen (O_2_) concentration falls below ∼ 0.08–0.256 mg L^–1^ (Lycus et al., 2017) denitrification becomes energetically favorable and is initiated through precisely coordinated regulation. An exception are aerobic denitrifiers which may utilize O_2_ and NO_3_^–^ simultaneously as electron acceptors, likely favorable in environments with fluctuating O_2_ concentrations and sufficient reduced carbon (Ji et al., 2015). Denitrification is known to occur in groundwater bodies (Korom, 1992). However, in some aquifers, none, or only little microbial available electron donors are present resulting in high dissolved O_2_ concentrations. Under these conditions, the intrinsic capacity for denitrification is low, whereby it will take years to decades (denitrification lag times) until the O_2_ is depleted and biotic NO_3_^–^ reduction commences (Wassenaar, 1995; Wild et al., 2018).

**FIGURE 1:**
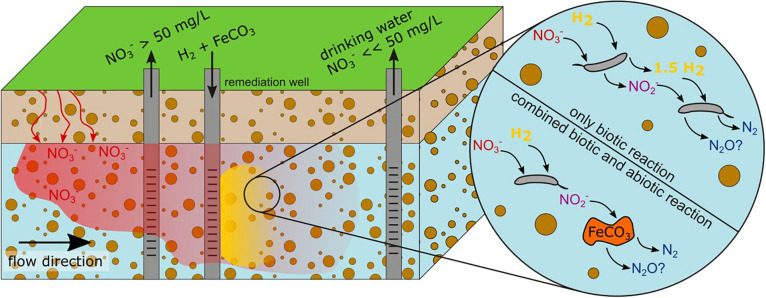
Concept of local *in situ* NO_3_^–^ remediation. The addition of H_2_ into an aquifer through a remediation well generates an anoxic H_2_-rich zone (yellow) where hydrogenotrophic denitrification is stimulated. Further downstream groundwater with NO_3_^–^ levels below the WHO recommended maximum of 50 mg L^–^^1^ could be obtained. Complementation with Fe(II)-containing minerals (e.g., siderite FeCO_3_), injected in the anoxic, H_2_-rich zone, could foster complete denitrification by abiotic reduction of the intermediate NO_2_^–^.

Creating conditions favoring denitrification and supplementation with an electron donor presents a strategy for small-scale *in situ* NO_3_^–^ remediation (Figure 1). Hydrogen (H_2_) was proven to be a promising electron donor in multiple NO_3_^–^ removal applications (Liessens et al., 1992; Chaplin et al., 2009; Karanasios et al., 2010). The risk of bio-clogging in aquifers due to H_2_ is lower compared to added dissolved carbon (Baveye et al., 1998) because the growth of autotrophic hydrogenotrophic denitrifiers is limited compared to heterotrophic denitrifiers in aquatic systems (Ergas and Reuss, 2001). Also, no by-products which would require further purification are formed during the oxidation of H_2_ to water (Lee and Rittmann, 2002). The possibility of local off-grid H_2_ production using wind or solar energy (Ulleberg et al., 2010; Onwe et al., 2020) provides another advantage. Drinking water sources and critical natural resources could be protected locally without building up elaborate infrastructure for a cost-effective long-term operation. Another way that allows for “clean” remediation of numerous environmental contaminants in groundwater is provided by bio-electrochemical systems which may supply bacteria directly with electrons, as discussed in the review by Cecconet et al. (2020).

Several studies have attempted to stimulate hydrogenotrophic denitrification in closed systems (Haugen et al., 2002; Chaplin et al., 2009; Kumar et al., 2018; Duffner et al., 2021), columns, bioreactors (Liessens et al., 1992; Chang et al., 1999; Haugen et al., 2002; Lee and Rittmann, 2002; Schnobrich et al., 2007) as well as *in situ* (Chaplin et al., 2009). However, transient NO_2_^–^ accumulation and/or incomplete denitrification has been reported in most of the batch and flow-through experiments. The H_2_ concentration was shown to be an influential factor determining complete denitrification because several flow-through experiments were able to reach an effluent NO_3_^–^ concentration below 1 mg NO_3_^–^-N/L and NO_2_^–^ concentration below detection limit by increasing the H_2_ pressure (Haugen et al., 2002; Lee and Rittmann, 2002; Schnobrich et al., 2007). Other chemo-physical parameters which influence the denitrification efficiency are pH (Li et al., 2017), carbon dioxide (CO_2_) availability, NO_3_^–^ and O_2_ concentrations, as well as the water flow velocity (Ergas and Reuss, 2001; Haugen et al., 2002). The pH optimum of hydrogenotrophic denitrification is between 7.6 and 8.6 (Karanasios et al., 2010). Increased pH above 8.6 can inhibit the process (Lee and Rittmann, 2003) and generally NO_2_^–^ accumulation increases with increasing pH (Lim et al., 2018). On the other side at pH 6.5 or lower the maturation of the nitrous oxide reductase is inhibited resulting in significant N_2_O accumulation (Liu et al., 2014). H_2_ injection may strip CO_2_ from groundwater, altering the CO_2_ availability and as a result also the pH. Thus, these parameters must be closely monitored. Additionally, the composition of the denitrifier community may determine whether denitrification is complete.

In the following sections, we will discuss the effects of H_2_ application on groundwater limited by atmospheric pressure and the influence of the hydrogenotrophic denitrifier community composition on the outcome of NO_3_^–^ remediation. These factors have been already discussed in literature as major drivers of hydrogenotrophic denitrification. We discuss their impact on NO_3_^–^ remediation in groundwater and how they can be controlled to foster complete denitrification. Additionally, we discuss combining the H_2_ amendment with the injection of Fe(II)-containing nano-sized minerals that stimulate abiotic NO_2_^–^ reduction to N_2_O and could thereby prevent NO_2_^–^ accumulation.

## Fostering Complete Denitrification – Hydrogen Concentration as a Major Trigger

The dissolved H_2_ concentration in groundwater is the most important factor determining hydrogenotrophic denitrification efficiency at a neutral pH. Chang et al. (1999) observed that at a dissolved H_2_ concentration below 0.1 mg L^–1^ the nitrate reductase is inhibited while the nitrite reductase is inhibited already below 0.2 mg L^–1^. As the nitrite reductase responds even more sensitive to low dissolved H_2_ concentrations than the nitrate reductase, NO_2_^–^ accumulation in groundwater because of H_2_ limitation is likely. Optimal H_2_ concentrations for complete nitrogen removal are between 0.4 and 0.8 mg L^–1^ H_2_ (Karanasios et al., 2011). Successful hydrogenotrophic denitrification with H_2_ concentration of 1.4 mg L^–1^, slightly below its maximum solubility of 1.6 mg L^–1^ (20°C, aqueous medium), have also been described in literature (Karanasios et al., 2010).

The dissolved H_2_ concentration in closed bottles with a headspace and a water phase can be determined easily as it is homogenous and proportional to the partial pressure of the headspace gas at a constant temperature according to Henry’s law. However, in settings with a continuous water flow, such as in bioreactors or in an aquifer, it is difficult to determine the dissolved H_2_ concentrations. Most H_2_ is consumed directly inside the biofilm that is growing on the H_2_ releasing membrane and additionally local conditions change continuously due to the groundwater flow (Haugen et al., 2002; Lee and Rittmann, 2002). The required H_2_ gas supply pressure to achieve locally sufficiently high dissolved hydrogen concentrations for complete denitrification also differs depending on the NO_3_^–^ and O_2_ concentrations, as well as the water flow velocity (Haugen et al., 2002; Lee and Rittmann, 2002). Increasing the H_2_ gas supply pressure was the determining factor in several continuous flow reactor experiments to achieve complete NO_3_^–^ and NO_2_^–^ reduction (Haugen et al., 2002; Lee and Rittmann, 2002; Schnobrich et al., 2007). To the best of our knowledge, in the only *in situ* experiment on hydrogenotrophic denitrification even an increase in the H_2_ lumen pressure from 1.68 atm to 2.36 atm could not resolve that only approximately half of the NO_3_^–^ was reduced to NO_2_^–^, but not further to N_2_O or N_2_ (Chaplin et al., 2009). As the lightest molecule, the diffusion coefficient of H_2_ in water is large, making it more difficult to obtain sufficiently high dissolved H_2_ concentration *in situ* compared to a closed system such as a bioreactor. Its high diffusion and the bacterial biofilm formation decrease the H_2_ mobility and its zone of influence needed for efficient NO_3_^–^ removal. The denitrification activity is known to be largest in a biofilm of medium thickness and decreases when the biofilm further thickens (Chu and Wang, 2013). Thus, a large area of gas exchange accommodating as many bacteria as possible would be advantageous. A promising method to deliver gas over a large surface area are hollow-fiber membranes, e.g., made of gas-permeable silicon tubes (Ho et al., 2001).

In conclusion, it is important to determine the required local dissolved H_2_ concentration at the membrane water interface under consideration of the water flow velocity, as well as dissolved O_2_ and NO_3_^–^ concentrations and the respective gas pressure needed to achieve this. Considering these difficulties of achieving sufficiently high dissolved H_2_ concentrations, initial *in situ* NO_3_^–^ remediation trials should focus on aquifers with high NO_3_^–^ pollution and innate low O_2_ concentrations so that only little H_2_ is utilized to react with the remaining O_2_.

## Fostering Complete Denitrification – The Role of Genomic and Phenotypic Plasticity of Denitrifiers

**FIGURE 2:**
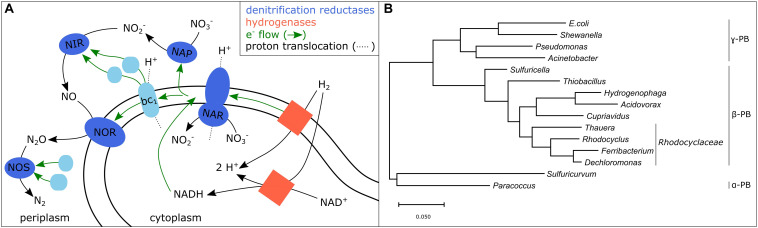
**(A)** Scheme of a potential electron transport chain of hydrogenotrophic denitrifiers based on Bothe et al. (2007) and Matassa et al. (2015). Hydrogenases, membrane-bound or soluble, transfer electrons to ubiquinone (not depicted) which delivers electrons within the cell membrane to the membrane bound denitrification reductases NAR and NOR and to the cytochrome bc_1_ complex. The later delivers electrons via two other redox-active proteins to the periplasmic denitrification reductases NAP, NIR, and NOS. **(B)** Phylogenetic tree of genera which include autotrophic bacteria detected in hydrogenotrophic denitrifying systems (PB, *Proteobacteria*; Karanasios et al., 2010; Kumar et al., 2018; Wu et al., 2018).

Disparity between the genetic potential and the observed denitrification phenotypes, for example lacking N_2_O reduction despite the presence of a nitrous oxide reductase gene (*nosZ)*, has been observed in several denitrifiers (Lycus et al., 2017). One possible explanation thereof is, that a functioning electron transfer coupled to proton translocation during denitrification (Figure 2A) requires several other proteins besides the reductases which ultimately influence the phenotypic outcome. These proteins include electron carriers, regulatory proteins, chaperonins, as well as proteins involved in metal processing, which together manage the maturation and finely coordinated regulation of the denitrification reductases (Philippot, 2002; Bothe et al., 2007). The genes encoding these proteins are arranged in gene clusters of which only few are conserved in all or most bacterial and archaeal genomes (Philippot, 2002). The disparities may have arisen due to several evolutionary drivers including horizontal gene transfer, convergent evolution of different structural types, as well as gene duplication and loss (Jones et al., 2008). The prediction of denitrification phenotypes based on genome sequences is in many cases still impossible, likely because of the divergence of gene cluster organization and the organization of those clusters in the genome. Additionally, the denitrification reductases compete for electrons from the electron transport chain (Albina et al., 2019). Some denitrification reductases are known to be stronger competitors (e.g., *narG*) than others (e.g., *napA*) (Gao et al., 2020) and the competition may even be additionally influenced by environmental factors such as pH (Albina et al., 2019).

Recent studies on heterotrophic pure cultures of denitrifiers (Bergaust et al., 2011; Liu et al., 2013; Lycus et al., 2017; Mania et al., 2020) revealed a vast phenotypic and genotypic diversity when investigating the difference in denitrification characteristics, termed “Denitrification Regulatory Phenotypes” (DRP). The phenotypic differences were visible in the O_2_ concentration at the onset of denitrification, the performed reduction steps, the electron flow rates to the individual denitrification reductases and resulting intermediate accumulation. Such differences were also observed in pure cultures of hydrogenotrophic denitrifiers (Vasiliadou et al., 2006). For example, while an *Acinetobacter* strain was accumulating 84.1% of the initial NO_3_^–^ as NO_2_^–^ with 1.37 mg L^–1^ dissolved H_2_, strains belonging to the genera *Acidovorax* and *Paracoccus* did not show any NO_2_^–^ accumulation (Vasiliadou et al., 2006).

Main taxa of the hydrogenotrophic denitrifier community, such as *Acidovorax*, *Paracoccus*, *Acinetobacter*, *Pseudomonas*, *Paracoccus*, *Rhodocyclus*, *Hydrogenophaga, Sulfuritalea*, and *Dechloromonas*, are well known from literature (Karanasios et al., 2010; Wu et al., 2018; Duffner et al., 2021) (Figure 2B). However, the results on DRPs show that phylogeny does not help to detect efficient hydrogenotrophic denitrifiers. Thus, the microbiological analysis must go beyond phylogeny and rather decipher the denitrification characteristics of hydrogenotrophic denitrifiers to identify intermediate accumulation and required parameters under which the former can be prevented. These required parameters include the previously stated such as minimal dissolved H_2_ concentration, maximum O_2_ concentration, dissolved CO_2_ availability, as well as the influence of biofilm formation and flow velocity. Once this data is available on widespread and efficient hydrogenotrophic denitrifiers, simple community analyses of site-specific hydrogenotrophic enrichment cultures could help to assess whether the native bacteria are able to perform complete denitrification and which conditions these bacteria require.

Generally, environmental conditions such as electron donor/electron acceptor interaction, which is highly affected by transversal dispersion in groundwater (Rolle et al., 2009), and dissolved carbon availability (Kraft et al., 2014) shape the bacterial community composition and the dominating metabolic pathways. In phylogenetically diverse microbial communities shifts in environmental conditions can change metabolic activities, while individual taxa do not have a notable influence. This is also true for denitrifying communities which have been described for example in bulk soil, where the degree of functional redundancy is high (Regan et al., 2017). Conversely, in less diverse bacterial communities, such as hydrogenotrophic denitrifiers (Karanasios et al., 2010; Duffner et al., 2021), the genotypic and phenotypic characteristics of individual taxa influence the dominating metabolic pathway more significantly, which makes the investigation of pure cultures even more relevant to understand hydrogenotrophic denitrification.

## Fostering Complete Denitrification – Combining Biotic Denitrification With Iron-Based Abiotic Nitrite Reduction

Microbial catalyzed reduction of oxidized nitrogen species is not the only environmental process of NO_3_^–^ remediation. Abiotic reduction of NO_2_^–^ by iron Fe(II), termed chemo-denitrification, is also known to occur under environmentally relevant conditions (Jones et al., 2015; Buchwald et al., 2016; Grabb et al., 2017). The reduction of NO_2_^–^ is catalyzed mainly by Fe(II) located on mineral surfaces while aqueous Fe(II) reacts much slower (Buchwald et al., 2016). Among the reactive Fe(II)-containing minerals are siderite (FeCO_3(s)_) (Rakshit et al., 2008), magnetite (Fe_3_O_4_) (Dhakal et al., 2013; Margalef-Marti et al., 2020), pyrite (FeS_2_), pyrrhotite (Fe_(1–x)_S), and biotite (Margalef-Marti et al., 2020). Fe(II)-containing minerals react rapidly with NO_2_^–^ and the reaction may be much faster than abiotic NO_3_^–^ reduction (Dhakal et al., 2013). This preference for NO_2_^–^ over NO_3_^–^ reduction makes Fe(II)-containing minerals a beneficial additive to counteract the accumulation of NO_2_^–^. Supplementation with Fe(II)-containing minerals alongside H_2_ injection thus presents a possible method to foster complete denitrification by sustaining NO_2_^–^ and NO reduction and leaving more dissolved H_2_ to microbial denitrification.

An important factor contributing to the reactivity may be the mineral size of Fe(II)-containing minerals. Nano-sized but not macro-sized magnetite lead to complete NO_3_^–^ reduction to N_2_ in an experiment by Margalef-Marti et al. (2020) due to greater surface area and Fe(II) availability. Additionally, nano-sized minerals have a wider range of distribution when injected into an aquifer. However, in order to prevent the exergonic oxidation of Fe(II) with dissolved O_2_ the nano-colloids should be injected directly with the H_2_ into the anaerobic plume *in situ*.

While reducing NO_2_^–^ accumulation, the addition of Fe(II)-containing minerals may increase accumulation of the gaseous intermediates NO and N_2_O, which was demonstrated by isotope measurements and the calculated isotopic offsets between NO_2_^–^ and N_2_O. These showed that much of the NO_2_^–^ consumed was not directly accounted for as N_2_O and likely accumulated as NO (Jones et al., 2015; Margalef-Marti et al., 2020). The complementation with H_2_ injection to stimulate hydrogenotrophic denitrifiers is therefore necessary and the effect of Fe(II)-containing minerals on the physiological characteristics of the underlying bacteria must also be examined. In this regard, Fe(II)-containing minerals may also be used as electron donors by numerous autotrophic denitrifiers (Otero et al., 2009; Jones et al., 2015; Hernández-del Amo et al., 2018; Margalef-Marti et al., 2020). As a result the abiotic reduction of NO_2_^–^ with Fe(II) may occur alongside microbial denitrification, the two processes are even interconnected (Melton et al., 2014) and may catalyze NO_3_^–^ remediation. In the study of Margalef-Marti et al. (2020) magnetite nano-particles alone rapidly reduced NO_2_^–^ to N_2_O but the addition of a microbial inoculum stimulated complete reduction to N_2_. Lithoautotrophic NO_3_^–^-dependent pyrite oxidation has been detected as the predominant denitrification process in a carbon-limited aquifer (Schwientek et al., 2008; Otero et al., 2009), thus the responsible bacteria are likely widespread. Even though NO_3_^–^-dependent Fe(II) oxidation is an energetically favorable metabolism at neutral pH, most NO_3_^–^ reducing Fe(II)-oxidizing bacteria require an additional electron donor or organic carbon for growth (Weber et al., 2006; Melton et al., 2014). These findings indicate that apart from the abiotic reduction, introducing a second electron donor to stimulate denitrification potentially increases the bacterial diversity making the enriched denitrifying community likely more resilient (Girvan et al., 2005).

Even though the injection of nano-sized particles to contaminated sites is already applied in some countries, concerns remain, including unknown long-term effects, transformation and ecotoxicity (Crane and Scott, 2012). For example, adverse effects of nano-sized iron on the biomass and activity of soil microbial communities under stress have been determined (Anza et al., 2019). Additionally, nano-sized particles or reaction products may react rapidly with sediment leading to clogging of the reactive zone and forcing the groundwater to bypass (Strutz et al., 2016). Therefore, before such nano-sized particles can be applied *in situ*, their long-term behavior in the investigated aquifer type must be determined.

## Summary and Outlook

Fostering complete hydrogenotrophic denitrification *in situ* can only be achieved by combining multiple approaches. First, it is important to determine and estimate a set of aquifer parameters in a NO_3_^–^ polluted groundwater to decide whether NO_3_^–^ remediation by H_2_ injection is feasible under the given hydrogeological conditions. These parameters include pH, organic and inorganic carbon contents, dissolved O_2_ concentrations and NO_3_^–^ concentrations, the groundwater flow velocity, potential biofilm formation, and the transversal dispersion. For example, when treating groundwater, pH shifts below 6.5 or above 8 may lead to an accumulation of NO_2_^–^ and make the NO_3_^–^ polluted aquifer unsuitable for bioremediation. Therefore, clean-up strategies of NO_3_^–^ polluted aquifers may only be sustainable in groundwater with sufficient inorganic carbon which is able to buffer pH changes. Second, hydrogen-enhanced denitrification requires an effective H_2_ transfer into the aquifer which must be adapted considering the previously stated parameters to ensure locally sufficiently high H_2_ concentrations. Third, the denitrification phenotypes of dominant hydrogenotrophic denitrifiers must be understood in depth. Hence the native bacterial community in the aquifer can be screened for complete hydrogenotrophic denitrifiers with little intermediate accumulation by amplicon sequencing approaches. Thus, one can determine whether the community is beneficial for complete denitrification or whether bacterial augmentation is necessary. Since these bacteria are generally low abundant in oxic groundwater, it is advised to enrich hydrogenotrophic denitrifiers under selective conditions before the screening.

Being a sequential process, it is difficult to avoid transient NO_2_^–^ accumulation during denitrification completely, especially *in situ*. When almost all dissolved O_2_ is reduced in groundwater an amendment with Fe(II)-containing minerals could thus aid the denitrifiers if periods of NO_2_^–^ accumulation occur and could potentially diversify the denitrifying community by providing an additional electron donor. Our analysis shows that remediation strategies of NO_3_^–^ polluted groundwater may be feasible in inorganic rich shallow groundwater systems that are characterized by low O_2_ concentrations, low organic carbon concentrations, but high NO_3_^–^ concentrations.

## Data Availability Statement

The original contributions presented in the study are included in the article/supplementary material, further inquiries can be directed to the corresponding author/s.

## Author Contributions

FE and MS developed the project idea and secured necessary funding to support the work of this manuscript. CD conceived and prepared the first draft of this manuscript. AW, SS, FE, and MS critically reviewed the draft. All authors contributed to the article and approved the submitted version.

## Conflict of Interest

The authors declare that the research was conducted in the absence of any commercial or financial relationships that could be construed as a potential conflict of interest.
